# The Effect of LC-MS Data Preprocessing Methods on the Selection of Plasma Biomarkers in Fed *vs.* Fasted Rats

**DOI:** 10.3390/metabo2010077

**Published:** 2012-01-18

**Authors:** Gözde Gürdeniz, Mette Kristensen, Thomas Skov, Lars O. Dragsted

**Affiliations:** 1 Department of Human Nutrition, Faculty of Life Sciences, University of Copenhagen, Rolighedsvej 30, 1958, Frederiksberg C, Denmark; Email: mkri@life.ku.dk (M.K.); ldra@life.ku.dk (L.O.D.); 2 Department of Food Science, Faculty of Life Sciences, University of Copenhagen, Rolighedsvej 30,1958, Frederiksberg C, Denmark; Email: thsk@life.ku.dk

**Keywords:** rat plasma, biomarkers, LC-QTOF, data pre-processing, MarkerLynx, MZmine, XCMS

## Abstract

The metabolic composition of plasma is affected by time passed since the last meal and by individual variation in metabolite clearance rates. Rat plasma in fed and fasted states was analyzed with liquid chromatography quadrupole-time-of-flight mass spectrometry (LC-QTOF) for an untargeted investigation of these metabolite patterns. The dataset was used to investigate the effect of data preprocessing on biomarker selection using three different softwares, MarkerLynx^TM^, MZmine, XCMS along with a customized preprocessing method that performs binning of *m/z* channels followed by summation through retention time. Direct comparison of selected features representing the fed or fasted state showed large differences between the softwares. Many false positive markers were obtained from custom data preprocessing compared with dedicated softwares while MarkerLynx^TM^ provided better coverage of markers. However, marker selection was more reliable with the gap filling (or peak finding) algorithms present in MZmine and XCMS. Further identification of the putative markers revealed that many of the differences between the markers selected were due to variations in features representing adducts or daughter ions of the same metabolites or of compounds from the same chemical subclasses, e.g., lyso-phosphatidylcholines (LPCs) and lyso-phosphatidylethanolamines (LPEs). We conclude that despite considerable differences in the performance of the preprocessing tools we could extract the same biological information by any of them. Carnitine, branched-chain amino acids, LPCs and LPEs were identified by all methods as markers of the fed state whereas acetylcarnitine was abundant during fasting in rats.

## 1. Introduction

In nutritional studies, blood samples are frequently collected in order to relate dietary conditions with metabolic markers. Blood may be obtained either in the fasted or postprandial state, depending on the hypothesis being tested. The fasting state, typically following an overnight fast, is considered to be more reproducible and can be defined as a baseline level for metabolic studies. However, imbalances in diet-dependent metabolism may not be detectable in the fasted state [1]. On the other hand, determination of the metabolic response in the extended postprandial state, which is the normal metabolic situation of human beings throughout the day, is more challenging as individual variability is high [2]. The basic metabolic rate varies roughly with surface area in mammals and an overnight fasting period in rats having an eight times higher rate of energy metabolism than humans may therefore represent a more extreme condition than overnight fasting in humans. A rat model may therefore be convenient to study the major differences between fasting and fed states, the latter defined as the state of rats following a normal *ad libitum* meal pattern. A rat model also offers full control of the food intake in the study subjects.

In this study, an untargeted metabolomics based approach to study the metabolic differences between rat plasma at fasted and fed states was performed. Metabolomics is defined as the process of monitoring and evaluating changes in metabolites during biochemical processes and has become an emerging tool to understand responses of cells and living organisms with respect to their gene expression or alterations in their lifestyles and diets of biochemical variation, during or after food intake [3].

A wide range of metabolites and other compounds can be detected in various biofluids by nuclear magnetic resonance (NMR) spectroscopy and mass spectrometry (MS). These approaches can be either untargeted through total data capture or highly targeted, such as measuring a large number of defined lipids. MS based instruments, with higher sensitivity compared to NMR [4,5], have become a widely used technique in metabolomics studies. Liquid chromatography (LC) coupled with time-of-flight (TOF) MS offers high resolution, reasonable sensitivity and improved data acquisition for complex sample mixture analyses. The system has served as a powerful tool in many other studies focusing on untargeted metabolic profiling of biofluids [6,7,8].

LC-MS analysis produces large amounts of data with complex chemical information. An important task is to arrange data in a way so that relevant information can be extracted. The complexity of LC-MS data brings out the concept of data handling which can be roughly summarized in two basic steps: data preprocessing and data analysis. *Data preprocessing* covers the methods to go from complex raw data to clean data. Raw data are comprised of retention times and mass to charge ratios of thousands of chemical compounds. Several software tools (commercial or freely available) have emerged for LC-MS data preprocessing. These tools typically include specific algorithms for the two key steps in data preprocessing, (1) peak detection and (2) alignment. Each software tool creates a list of peaks denoted by a specific mass and retention time. Each entry has a signal intensity denoting peak height or area. Alignment corrects retention time and mass differences across samples so that a peak, considered as one chemical compound, is represented by the same mass and retention time across all samples. The peak detection and alignment result in a data table providing the detected peaks across samples which can be denoted as clean data. All of these tools aim to provide high speed, automated data preprocessing. The basic principles of the many LC-MS data preprocessing software tools have recently been summarized [9,10]. To be able to obtain high efficiency in data preprocessing, the software tool employed should have the parameter settings required to match the structure of the specific dataset.

Existence of various data preprocessing tools brings out concerns about what are the characteristics of the software tools and what are the pros and cons of their algorithms. There are some studies attempting to define quality parameters for comparison of peak detection [11,12] or alignment [13] algorithms of different data preprocessing tools, but a direct comparison of the overall performance of the most commonly used data preprocessing tools has not so far been attempted. The question to be addressed in this study is whether there is agreement between the biological information as represented by the biomarkers extracted by preprocessing the same dataset with different data preprocessing methods. Therefore we compare here the potential biomarkers extracted from the current small dataset using four different softwares for preprocessing; (1) MarkerLynx^TM^ (MassLynx (Waters, Milfold, MA, USA)); (2) MZmine [14]; (3) XCMS [15,16] and (4) a customized method that is implemented in MATLAB (The Mathworks, Inc., MA, USA). MarkerLynx^TM^ is a commercial software whereas XCMS and MZmine are freely available software tools. The customized method included *m/z* binning and retention time collapsing which can be considered as a more old-fashioned method for LC-MS data preprocessing. The applicability of this method for LC-MS data has been evaluated in other studies [17] but an extensive comparison with other approaches has not been published previously.

Thus, in this study the UPLC-QTOF profiles of rat plasma collected in the fasted and fed states were analyzed for two different purposes: (1) to investigate the effect of different data preprocessing tools on biomarker selection; and (2) to interpret the biology behind the biomarkers identified for the two states.

## 2. Results and Discussion

### 2.1. Comparison of Data Preprocessing Methods

The number of features obtained from each preprocessing method is given in the Supplementary information 3. We succeeded in extracting a similar number of features with optimized parameter settings (positive or negative), except for the custom method in negative mode where we have an approximate doubling compared with the other software tools.

**Figure 1:**
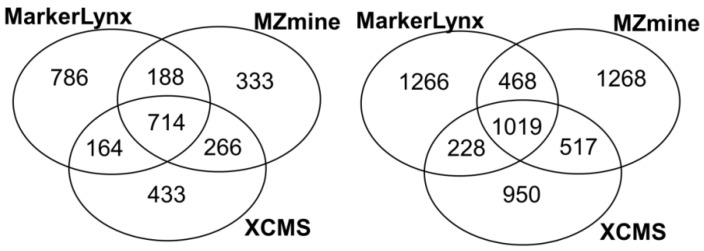
Venn diagrams illustrating the number of common and method specific features extracted from three software tools (right: positive mode; left: negative mode).

Primarily, common and unique extracted features from three different softwares were illustrated in Figure 1. We found 37%–46% of the features extracted by each software to be in common. Rauf *et al.* [16] found higher number for common features (46%–52%) from leaf and seed extracts comparing MZmine and XCMS (centWave) peak detection algorithms. The difference can be the result of more complex nature of plasma samples compared to plant extracts.

**Figure 2:**
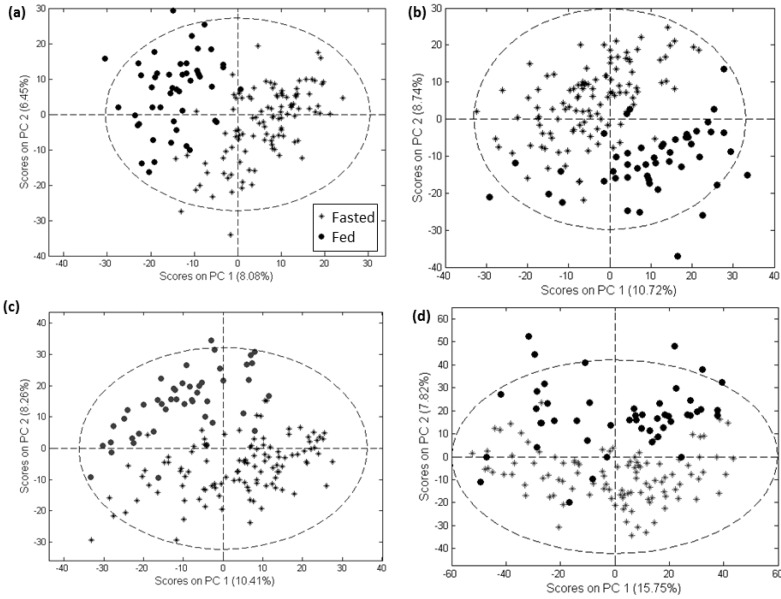
PCA scores plots of negative mode data processed with MarkerLynx (**a**), MZmine (**b**), XCMS (**c**) and customized methods (**d**).

All three software tools and the customized method employed here were able to produce a feature set showing substantial separation of samples from the fasted and fed states in a PCA scores plot (Figure 2 for negative mode data and Supplementary information 5 for positive mode data).

PLSDA model of each preprocessed data on independent test sets provided an average classification error rate of 0–0.02 (Supplementary information 6) indicating that all models resulted in good classification performance. The classification error rates were very similar for datasets obtained from different data preprocessing methods. On the other hand, the average classification error rates of datasets where classes were permuted were calculated as 0.49–0.51, corresponding to misclassification of half of the samples, which is an expected value for permuted data [18]. None of the 2,000 permutations had classification error lower than 0.00–0.02, indicating original fasted *vs.* fed discrimination was significant. Histograms of permutation test are given in Supplementary information 7.

As previously mentioned, autoscaling is applied in this study to detect possible variation between two states for any feature, regardless of its concentration. Nevertheless, autoscaling complicates variable selection as it gives the same chance to all peaks to influence the PLSDA model, and the decision of a regression coefficient cut-off value for selection of important features becomes difficult. Hereby, we decided to select of 25 features only but there is no proof to say the feature with 26th highest regression coefficient was not a potential biomarker. Thus, the 25 markers from each method and their various ranks from other softwares were included in Table 1 and Table 2 for the negative and positive modes, respectively. While there is no way to say which software is the more correct, the consequence of the differences observed here is that there is no basis for putting too much emphasis on the rank in PLS-DA methods. Howeverin many metabolomics studies, PLS-DA regression coefficients or VIP cut-offs have commonly been employed for marker selection, even without the rigorous iteration used here.

**Table 1:** Retention times and measured masses of the markers obtained from MarkerLynx, MZmine, XCMS and custom data processing of negative mode data that contributed most to the separation of samples in fasted and fed states.

NO	RT (min)	Measured *m/z*	MX Rank	MZ Rank	XCMS Rank	Custom rank	Group	Suggested Compound	Adduct	Monoisotopic mass
1	0.64	105.02	57	17	14	194	fed	U1		
2	0.82	116.07	91	26	17	507	fed	U2		
3	1.15	180.06	67	28	21	27	fed	U3		
4	1.15	383.12	40	80	25	624	fed	U3		
5	1.36	59.01	21	34	9	7	fasted	3-hydroxybutanoic acid F		104.0473
6	1.36	260.00	49	68	nd	22	fasted	3-hydroxybutanoic acid F		104.0473
7	1.37	229.07	20	35	nd	72	fasted	3-hydroxybutanoic acid A	[2M+Na-H]	104.0473
8	1.37	103.04	39	15	nd	20	fasted	3-hydroxybutanoic acid	[M-H]	104.0473
9	1.37	261.18	1424	nd	18	14	fed	Isoleucine	[2M-H]	131.0946
10	1.37	130.09	25	nd	24	65	fed	Isoleucine	[M-H]-	131.0946
11	1.80	178.05	nd	22	nd	166	fed	U4		
12	1.88	134.06	14	9	6	40	fasted	Hippuric acid * F		179.0582
13	1.88	178.05	15	7	4	116	fasted	Hippuric acid *	[M-H]	179.0582
14	2.02	344.10	383	nd	222	12	none	U5		
15	2.46	365.07	3	6	nd	43	fed	U6		
16	2.46	623.36	8	nd	3	94	fed	U6		
17	2.46	343.08	2	2	1	6	fed	U6		
18	2.47	623.87	4	nd	nd	16	fed	U7		
19	3.00	185.12	793	23	77	284	fed	U8		
20	3.50	505.30	1833	nd	nd	10	none	U9		
21	4.11	586.31	nd	13	nd	13	fed	LPC(20:5)	[M+FA-H]	541.3168
22	4.12	309.20	1	10	7	1802	fed	LPC(20:5) F		541.3168
23	4.15	452.28	22	30	22	1006	fed	LPC(14:0) F		467.3012
24	4.16	512.30	17	21	19	45	fed	LPC(14:0) A	[M+FA-H]	467.3012
25	4.16	979.60	19	nd	nd	33	fed	LPC(14:0) A	[2M+FA-H]	467.3012
26	4.17	502.29	13	11	nd	25	fed	LPC(18:3) F		517.3168
27	4.18	562.31	5	8	51	17	fed	LPC(18:3)	[M+FA-H]	517.3168
28	4.18	818.50	16	nd	nd	1672	fed	U10		
29	4.18	526.30	11	19	11	912	fed	LPC(20:5) F		541.3168
30	4.19	586.31	7	18	8	13	fed	LPC(20:5)	[M+FA-H]	541.3168
31	4.23	563.32	nd	nd	13	15	fed	U11		
32	4.34	476.28	23	1	nd	1	fed	2-acyl LPC(18:2) F		519.3325
33	4.35	564.33	10	12	nd	3	fed	2-acyl LPC(18:2)	[M+FA-H]­	519.3325
34	4.35	504.31	147	3	nd	2	fed	2-acyl LPC(18:2) F		519.3325
35	4.35	578.30	nd	5	nd	35	fasted	U12		
36	4.36	632.33	120	25	nd	113	fed	U13		
37	4.38	281.25	33	nd	15	nd	fasted	U14		
38	4.43	476.28	105	4	2	1	fed	1-acyl LPC(18:2) F		519.3325
39	4.44	168.35	6	nd	nd	1512	fed	1-acyl LPC(18:2) F		519.3325
40	4.44	995.59	60	nd	nd	4	fed	1-acyl LPC(18:2) F		519.3325
41	4.44	168.63	18	nd	nd	170	fed	1-acyl LPC(18:2) F		519.3325
42	4.44	504.31	65	14	32	2	fed	1-acyl LPC(18:2) F		519.3325
43	4.45	457.10	12	nd	561	2332	fasted	U15		
44	4.45	564.33	32	31	20	3	fed	1-acyl LPC(18:2)	[M+FA-H]	519.3325
45	4.45	335.40	nd	nd	nd	8	none	none		
46	4.45	335.70	nd	nd	nd	9	none	none		
47	4.45	477.28	nd	nd	nd	21	fed	1-acyl LPC(18:2)^ iso1^		
48	4.45	564.10	nd	nd	nd	23	none	none		
49	4.45	565.34	nd	nd	nd	5	fed	1-acyl LPC(18:2)^ iso2^		
50	4.45	587.30	nd	nd	nd	11	none	none		
51	4.45	996.59	nd	nd	nd	19	fed	1-acyl LPC(18:2)^ iso3^		
52	4.50	552.33	24	46	63	320	fed	U16		
53	4.62	452.28	48	55	23	1006	fasted	U17		
54	4.65	566.35	374	24	nd	138	fed	1-acyl LPC(18:1)	[M+FA-H]	521.3481
55	4.73	478.29	9	16	12	18	fed	LPE(18:1) *	[M-H]	479.3012
56	4.88	445.33	76	20	10	1206	fasted	U19		
57	5.14	277.22	85	106	5	98	fasted	Gamma-Linolenic acid *	[M-H]	278.2246
58	5.22	338.30	100	nd	nd	24	none	U20		
59	5.38	279.23	145	nd	16	177	fasted	Linoleic acid *	[M-H]	280.2402

MX: MarkerLynx; MZ: MZmine; ‘U’, Unidentified compound; A: Adduct; F: Fragment *, identity confirmed with authentic standards; ‘nd’, not detected by the software peak-finding algorithm.

**Table 2:** Retention times and measured masses of the markers obtained from MarkerLynx, MZmine, XCMS and custom data processing of positive mode data that contributed most to the separation of samples in fasted and fed states.

NO	RT (min)	Measured *m/z*	MX Rank	MZ Rank	XCMS Rank	Custom rank	Group	SuggestedCompound	Suggested Adduct	Monoisotopic mass
1	0.53	112.11	nd	12	13	301	fasted	U1		
2	0.57	730.70	276	nd	nd	25	fasted	U2		
3	0.61	103.04	46	nd	19	2901	fed	L-Carnitine *F		161.1052
4	0.61	102.09	1368	nd	21	481	fed	L-Carnitine *F		161.1052
5	0.61	162.11	31	41	10	10	fed	L-Carnitine *	[M+H]	161.1052
6	0.66	70.07	12	11	25	22	fed	D-proline *F		115.0633
7	0.66	116.07	13	14	12	11	fed	D-proline *	[M+H]	115.0633
8	0.86	130.09	24	521	44	838	fasted	U3		
9	0.90	144.10	23	nd	16	455	fasted	L-Acetylcarnitine*F		203.1158
10	0.90	204.12	28	18	6	8	fasted	L-Acetylcarnitine*	[M+H]	203.1158
11	0.90	145.05	21	13	11	41	fasted	L-Acetylcarnitine*F		203.1158
12	1.17	248.15	49	23	7	38	fasted	U4		
13	1.64	231.12	nd	100	1	649	fasted	U5		
14	1.90	105.03	1	17	2	78	fasted	Hippuric Acid*F		179.0582
15	1.90	77.04	3	19	3	578	fasted	Hippuric Acid*F		179.0582
16	2.23	316.21	19	46	nd	179	fasted	U6		
17	2.42	899.43	nd	nd	nd	17	fed	U7		
18	2.42	287.20	nd	nd	nd	1	fed	U7		
19	2.42	286.20	7	3	50	4	fed	U7		
20	3.42	536.34	35	nd	nd	24	fed	U8		
21	3.49	158.16	338	222	63	19	fasted	U9		
22	4.11	542.33	16	16	nd	21	fed	LPC(20:5)	[M+H]	541.3168
23	4.12	564.31	nd	15	nd	43	fed	LPC(20:5) A	[M+Na]	541.3168
24	4.16	312.03	151	nd	17	2659	fed	U10		
25	4.16	468.31	20	24	23	15	fed	LPC(14:0)	[M+H]	467.3012
26	4.19	540.31	25	64	nd	47	fed	LPC(18:3) A	[M+Na]	517.3168
27	4.19	518.33	15	6	81	62	fed	LPC(18:3)	[M+H]	517.3168
28	4.23	445.40	nd	nd	nd	12	fasted	octadecanoylcarnitine^Iso^		
29	4.23	444.37	18	33	47	33	fasted	octadecanoylcarnitine		
30	4.35	337.28	9	9	5	57	fed	2-acyl LPC(18:2) F		519.3325
31	4.35	520.34	6	1	nd	2	fed	2-acyl LPC(18:2)	[M+H]	519.3325
32	4.36	542.33	4	2	nd	21	fed	2-acyl LPC (18:2) A	[M+Na]	519.3325
33	4.36	819.96	22	nd	nd	950	fed	U11		
34	4.36	502.33	nd	10	nd	28	fed	2-acyl LPC(18:2) F	[M+Na]	479.3376
35	4.42	566.32	1024	2058	15	50	fasted	U12		
36	4.42	844.47	219	233	20	1312	fasted	U13		
37	4.44	519.90	nd	nd	nd	18	fed	U14		
38	4.44	521.35	nd	nd	nd	5	fed	1-acyl LPC(18:2) ^Iso1^	[M+H]	519.3325
39	4.45	523.35	nd	7	nd	89	fed	1-acyl LPC(18:2)^Iso2^	[M+H]	519.3325
40	4.45	519.70	316	nd	nd	7	fed	U15		
41	4.45	997.64	14	20	9	3	fed	1-acyl LPC(18:2) A		519.3325
42	4.45	819.97	2	21	835	950	fasted	U16		
43	4.45	520.34	8	4	18	2	fed	1-acyl LPC(18:2)	[M+H]	519.3325
44	4.45	998.64	30	nd	nd	6	fed	U17		
45	4.45	460.29	59	54	14	612	fed	1-acyl LPC(18:2) F		519.3325
46	4.45	520.10	nd	nd	nd	13	none	U18		
47	4.45	520.90	nd	nd	nd	23	none	U18		
48	4.45	521.55	nd	nd	nd	20	none	U18		
49	4.45	521.80	nd	nd	nd	16	none	U18		
50	4.45	807.97	5	8	4	2664	fed	U19		
51	4.63	949.64	34	25	48	85	fasted	U20		
52	4.64	454.30	32	22	22	1425	fasted	U20		
53	4.65	975.70	76	nd	nd	14	fed	U21		
54	4.65	522.36	10	nd	nd	70	fed	2-acyl LPC(18:1) *	[M+H]	521.3481
55	4.65	339.29	17	5	8	573	fed	2-acyl LPC(18:1) *F		
56	4.68	520.34	11	nd	24	2	fed	U22	[M+H]	519.3325

MX: MarkerLynx; MZ: MZmine; ‘U’, Unidentified compound; A: Adduct; F: Fragment *, identity confirmed with authentic standards; ‘nd’, not detected by the software peak-finding algorithm.

### 2.2. Custom Method *vs.* Software Tools

The algorithm of the custom preprocessing method differs from the others by not having any peak detection and alignment steps. It can therefore be considered as more independent, albeit more primitive and simple.

**Figure 3:**
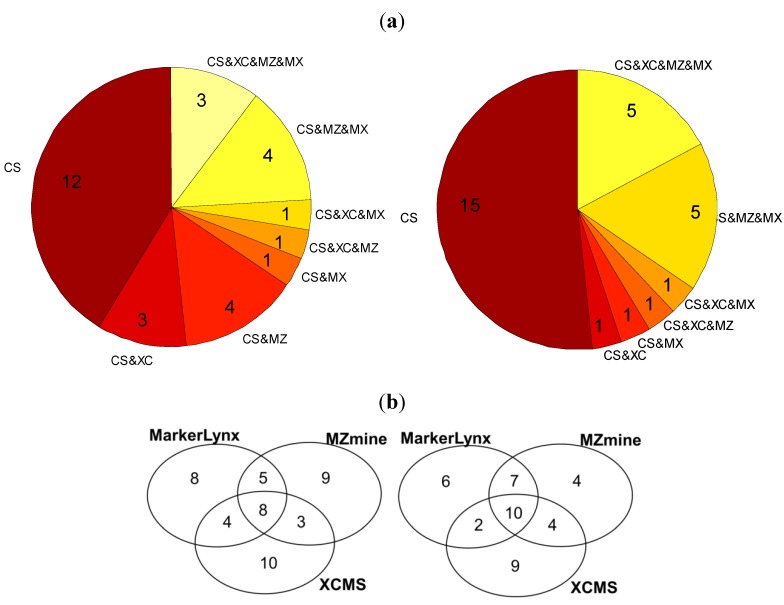
(**a**) Pie chart illustrating the number of custom data preprocessing markers that are unique and that are detected as markers by the other software tools (CS:Custom, MZ:MZmine, XC:XCMS, MX:Markerlynx); (**b**) Venn diagrams illustrating the number of common and method specific markers extracted from three software tools (right: positive mode; left: negative mode).

We compared first the *m/z* bins selected by the custom method with the markers from the three dedicated softwares (Figure 3a). Out of 25, only five of them were common for all data preprocessing tools in the positive mode and three in the negative. On the other hand, 48% (positive mode) and 58% (negative mode) of the *m/z* bins were identified also as markers by at least one of the software tools.

**Figure 4:**
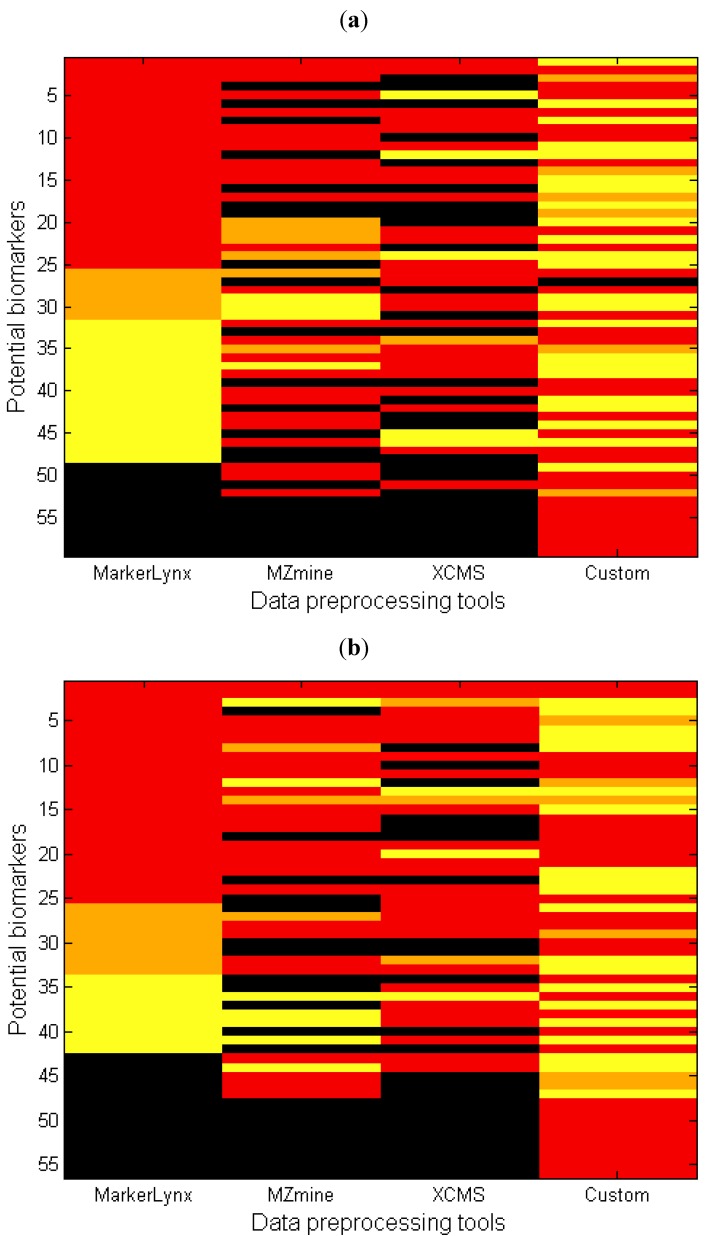
Heatmap comparing the importance of each marker based on four different data preprocessing tools for (**a**) negative and (**b**) positive mode data. Each row represents the lowest value rank of a metabolite for four different methods (Table 1, 3rd column). The markers were sorted in ascending order based on the rank obtained with MarkerLynx (red: rank 1–25; orange: rank 26–50; yellow: rank > 50; black: not detected).

Another perspective in the comparison of different data preprocessing methods is illustrated in Figure 4 where, each row represents the rank of one marker from Table 1 (columns 4–7) for all four different data preprocessing methods. The first impression from this figure may be that the number of black regions (undetected peaks) might seem alarmingly high for some of the methods. It is important here to state that the custom data preprocessing leads to a number of false positives. The major causes of false positives are splitting of analytes into two adjacent bins or chromatographic collapsing.

An additional point from Figure 4 is the large area of yellow regions for the custom method, which presents markers detected with higher than 50 as rank in PLS-DA. This is explained mainly by retention time collapse causing peaks to be added with other peaks having the same mass but different retention time. For instance, the chromatogram of *m/z* bin = 820 as illustrated in Supplementary information 8 includes two peaks. The sample track signals of the peak at retention time = 4.32–4.38 (No. = 33, Table 2) is higher in the fed state while the peak with retention time = 4.4–4.48 38 (No. = 42, Table 2) is higher in the fasted state, indicating that they are actually markers. As these two different peaks are in the same *m/z* bin, the retention times collapsing leads to the loss of these markers. In other cases a small peak representing a marker is added with a larger one without marker characteristics thereby diluting the effect so that the bin escapes selection.

### 2.3. Comparison of the Dedicated Software Tools

Further comparison of the 25 markers for positive and negative mode data from each of the three dedicated software tools is illustrated in two Venn diagrams (Figure 3b). In general these three tools seem to have 8–10 markers in common among the selected 25 markers detected in the negative and positive mode (Figure 3b). There is a trend towards a larger difference between XCMS and each of the other methods in the pairwise comparisons. So all of the data preprocessing methods seem to miss out potentially important markers observed to be ranked among the top-25 markers by the other methods. In fact, only 8–10 markers would be observed to be in common if three different research groups were to investigate the same biological phenomenon using different softwares for data preprocessing, provided they had recorded similar LC-MS data. There are three possible explanations of the differences between detected markers:

(1) The marker is not included in the feature list of the other softwares. The potential cause is differences between peak detection algorithms. The number of detected features is different as shown in Figure 1. This condition is illustrated by Figure 4 as black regions.
(2) The marker is detected but the peak height assignment was not the same among software tools, which did not result in significant difference between fasted and fed states. One reason of this is shown in the next section as influence of gap filling. This condition is illustrated as yellow in Figure 4.
(3) The data analysis method affected the marker selection. This was discussed as an effect of autoscaling previously. This condition is illustrated by orange in Figure 4.

Additional differences might be caused by optimization of parameter settings and other factors from the metabolomics experiment. The loss of information and potential introduction of noise from feature selection by a single preprocessing method would therefore seem to be a potential source of error in metabolomics.

### 2.4. The Influence of Gap Filling

An important drawback for MarkerLynx^TM^ is that it does not contain any gap filling algorithm resulting in many zero values in the final extracted feature set. Zeros may obscure the later data analysis step and may result in incorrect grouping of ‘effect markers’ and ‘exposure markers’, because ‘true’ zeros as well as smaller and larger peaks missed by the algorithm are given the same zero value [19]. Consequences of this lacking gap filling algorithm is illustrated with two real cases. In the first case, MarkerLynx^TM^ algorithm records the signal of some samples from the group with a lower signal as zero, thereby increasing the differences between groups and the chance that the feature is selected as a marker. For instance, marker number 42 (Table 2) has rank 2 for MarkerLynx^TM^ whereas it came out with higher ranks by the others (Supplementary information 6) due to this phenomenon. In the second case, the signal of some samples recorded as zero while those samples belong to the group with higher signal. In this way, the true difference between the two groups was deflated and those markers had higher rank number (lower importance) with MarkerLynx^TM^. Many examples (Supplementary information 9) of this situation is observed, particularly in the negative mode data where the signal intensity is generally lower, thereby explaining the large yellow region for MarkerLynx^TM^ in Figure 4a.

Another observation particularly in Figure 4a is that MarkerLynx^TM^ has fewer black regions, meaning very few undetected peaks and several markers that are detected by MarkerLynx^TM^ but not by the other two softwares. Since the total number of features obtained from preprocessing the data was similar for all three softwares, one possible explanation could be the differences in the filtering step. The 80% rule applied to the MarkerLynx^TM^ dataset differs from that of the others by retaining features with many non-zero observations in at least one sample group. The filtering algorithm of MZmine does not allow the user to define the filter for each sample group. By filtering away features with many zeros, there is a risk of removing perfect markers that appear only in one of the sample groups. Therefore the filter has to be set to no more than 80% of the number of observations in the smallest sample group in order to be equivalent to the 80% rule. Another possible reason could be the differences between the peak detection algorithms. MarkerLynx^TM^ provides an automated peak detection algorithm whereas many parameters are user-defined for the others. Although we optimized the selection of parameters carefully by testing several settings, we cannot rule out that better overlap could have been obtained with a different parameter set.

### 2.5. Software Preprocessing Settings

The number of detected peaks depends very much on the data preprocessing settings of each software algorithm. Although we attempted to attain the largest possible similarity in the preprocessing parameters of MarkerLynx^TM^, MZmine and XCMS, we were aware that it is not possible to obtain exactly the same results, since each method is based on different algorithms. To illustrate this point, we preprocessed the data with MZmine using less conservative settings for many peak detection parameters and constructed the heat map again, leading to a new pattern much more similar to XCMS (figure not shown). So, in reality, it may be possible to obtain similar patterns, at least with MZmine and XCMS where gap filling is available, depending on their individual parameter settings.

In this study the contrasts between the fasted and fed states were very clear, whereas such strong contrasts may not be seen in many other metabolomics studies. Improper settings of data preprocessing parameters may therefore obscure the extraction of relevant information, and several settings and/or softwares should be applied. Proper settings are based on careful inspection of raw data as well as insight into the functionalities of software parameters. It could seem like an appealing option to allow a much larger number of peaks by being less conservative with many peak detection parameters. However, the consequence of detecting many peaks will be the inclusion of more noise and will complicate not only the alignment but also the data analysis step for the detection of biomarkers.

MarkerLynx^TM^ and MZmine are both user friendly tools for users who do not want to go into R, MATLAB, or similar programming tools. Preprocessing data with MarkerLynx^TM^ requires just a few user-defined settings. However the software does not provide any possibility for checking the success of any data preprocessing step. In comparison, MZmine provides a powerful visualization side that can be considered as quite useful for tuning the settings. Algorithms for visualization of peak detection results are also included in the XCMS package in R.

### 2.6. Biomarker Patterns

Three patterns are immediately visible for markers of the fed state in Table 1 and Table 2. The first of these is the presence of sets of isomers having very similar masses but slightly different retention times, indicating that some specific groups of isomers are typical markers. The slight mass difference may be attributed to the mass accuracy of the instrument. Some examples are clusters at 512.29, 478.29 and 590.35 in the negative mode, and at 468.32, 520.34, and 522.36 in the positive mode. In many cases the earlier eluting isomeric form was not detected in the XCMS preprocessed dataset, possibly because they are much smaller peaks. Considering the parameters set while preprocessing the data with XCMS (Supplementary information 10), additional filtering or a too high *bw* parameter (for setting the RT shift) might be the cause of not detecting those peaks. Furthermore, these patterns are always spotted with the custom data preprocessing as they were included into the same *m/z* bin, thereby intensifying their relative importance. As can be seen from Table 1 and Table 2, the possible isomers were therefore given the same rank for the custom data preprocessing.

Another pattern in the marker sets is the presence of peaks with mass differences corresponding to 2 or 4 hydrogen atoms but with different retention times. These pairs are observed in both modes (e.g., 476/478, 562/564/566 in the negative mode, and 506/508 or 520/522 in the positive, Table 1 and Table 2). These clusters and patterns are all observed for compounds with retention times in the same (unpolar) range pointing towards a series of lipids with varying levels of saturation (2 for each double bond).Similar patterns can also be observed for changes in chain lengths (+26 for adding –CH=CH–) as the underlying biomarkers. 

Pattern recognition therefore identified lipids as potential discriminative markers between plasma samples collected at fasted and fed states. This confirms an expected finding and further identification of some of the lipids as well as some of the more polar peaks was therefore perused.

### 2.7. Biomarkers of Fasted and Fed State

Most of the masses belonging to the lipid-related patterns and clusters in the positive mode fit with the masses expected for positively charged lysophosphatidylcholines (LPCs) of varying chain lengths and degrees of saturation. LPC is a plasma lipid that has been recognized as an important cell signaling molecule and it is produced by the action of phospholipases A1 and A2, by endothelial lipase or by lecithin-cholesterol acyltransferase (LCA).LCA has a well-known function in catalyzing the transfer of fatty acids from phosphatidylcholine to free cholesterol in plasma for the formation of cholesteryl esters [20]. In the rat, the LPCs with more saturated acids are formed mainly in the plasma whereas unsaturated LPC is formed from PCs in the liver. We observe here a mixture of both saturated and unsaturated LPCs, indicating that the source may be dual. The cytolytic and pro-inflammatory effects of LPCs are well-known so their level is closely regulated. However, in blood plasma the LPCs form complexes with albumin and lipoproteins, especially LDL, and are therefore not as likely to cause direct cell injury [21]. Another action of LPCs seems to be related to increased insulin resistance [22]. A slow clearance of postprandial lipids is known to be a risk factor for diabetes but the LPCs might be a lipid fraction contributing more strongly to this action. It is interesting in this context to note that Kim *et al.* identified LPCs as the major discriminative compounds of plasma species separating fasting plasma from obese/overweight and lean men [7]. They reported lower levels of saturated LPCs and higher level of unsaturated LPCs in the plasma of lean as compared to obese or overweight men. We found a similar profile here in lean rats. The unsaturated LPCs have also been found to pass the blood-brain barrier and to be important vehicles for delivering unsaturated lipids to the brain [23]. We speculate that the high level of unsaturated LPCs in the postprandial state of healthy individuals might be part of the satiety signaling system which is malfunctioning in obesity.

The LPCs appear usually in two isomeric forms, as 1-acyl or 2-acyl LPCs. The true separation of isomeric groups of LPC(18:1) in a fed state plasma sample is illustrated in Supplementary information 11. These isomers were unstable and spontaneously isomerized positionally, as also recognized in 1-acyl authentic standards of LPC and LPE(18:1), where 9% of the authentic standard was detected as the peak belonging to the 2-acyl form. For the confirmation of the 2-acyl LPC form, standards of PC and PE(16:0/18:1) were hydrolyzed by phospholipase A1. In addition to the 2-acyl LPC and LPE(18:1) we observed that 7% of the acyl group had spontaneously migrated to the 1-acyl position (Supplementary information 11). Croset *et al.* studied the significance of positional acyl isomers of unsaturated LPCs in blood [24]. They concluded that 50% of PUFA was located at the 2-acyl position where they are available for tissue uptake, and that they can be re-acylated at the 1-acyl position to form membrane phospholipids. 

With the applied methodology we would only be able to extract the more polar lipids and detect lipids with *m/z* below 1,000 daltons. Therefore, we cannot conclude here that the LPCs, LPEs and free fatty acids are the major discriminative lipid species. Lipidomics studies have previously reported less polar lipid classes which may have *m/z* above 1,000 daltons, such as PCs, sphingomyelins and triacylglycerols as potentially reflecting the time since last meal [25,26]. With our current method, we were able to identify PCs but they were not discriminative in this study, possibly due to incomplete extraction.

A group of carnitine based compounds was also detected as markers in the positive mode data. The main function of carnitine is to assist the transport and metabolism of fatty acids in mitochondria, where they are oxidized as a major source of energy [27]. In the plasma samples from the fasting state, the level of L-carnitine was found to be lower whereas acetyl-L-carnitine was higher. During fasting an elevated concentration of acetyl coenzyme A favors the production of acetyl-L-carnitine and the ketone body, 3-hydroxybutanic acid [28], and these were identified as characteristic markers for the fasting state.

Two of the amino acids, isoleucine and proline, were found to be strongly discriminating between the fed and fasted states. Isoleucine belongs to the group of branched-chain amino acids which have been implicated in altered protein catabolism, insulin resistance and obesity [29,30]. However, leucine may have contributed to the signal since separation by our current UPLC-method was not efficient. It seems therefore that isoleucine, and possibly other specific amino acids, may be markers of recent food ingestion and decrease with fasting.

Many adduct or daughter ions were also observed among our markers as shown in Table 1 and Table 2. In many cases, different adducts or fragment ions of the same metabolite may emerge with a higher or lower rank than the parent ion, and this is an important cause of differences in the ranking orders between the preprocessing softwares. So at the metabolite level, the differences between the preprocessing methods are actually much smaller. To illustrate the higher concordance at the metabolite level, we established a new rank for each metabolite (giving each metabolite the lowest rank value from among its representative adducts, fragments or isomers). The unidentified features were considered as representing the same metabolite as long as they are within the range of 0.02 min retention time window. The metabolite ranks of different methods are represented in Supplementary information 12, which illustrates that the rank patterns were much more similar between different methods at the metabolite level than at the feature level (Figure 4). Thus, it seems reasonable to conclude that different data preprocessing methods employed in this study provide around 50% common markers, but the agreement is actually much higher at the metabolite level since different markers (adducts or fragment ions) selected from the different preprocessing softwares represent the same metabolites.

The observation that all these related ions come up with low rank numbers, *i.e.*, high importance, and that their low ranks are shared between positive and negative modes as in this study strengthens not only the confidence in the identification step but also in our variable selection method.

## 3. Experimental Section

### 3.1. Animal Study and Sample Collection

Eighty male Fisher 344 rats (4 weeks old) were obtained from Charles River (Sulzfeld, Germany). The animals had a one week run-in period to adapt to the standardized diet. The rats were subsequently randomized into five groups of 16 rats, each with equal total body weights and then fed five different diets which were all nutritionally balanced to give exactly the same amounts of all important macro- and micronutrients [31]. After 16 weeks, all rats were sacrificed by decapitation after CO_2_/O_2_ anesthesia. Before sacrifice, 56 of the animals had fasted for 12 h and 24 of the animals were given access to food up until termination. Blood samples were collected immediately after sacrifice directly from the *vena jugularis* into a heparin coated funnel drained into 4 mL vials containing heparin as an anticoagulant. The blood was centrifuged at 3,000 g, 4°C for 10 min. The plasma fraction was aliquoted into 2 mL cryotubes and stored at −80°C until further processing. The animal experiment was carried out under the supervision of the Danish National Agency for Protection of Experimental Animals.

### 3.2. Plasma Preprocessing and LC-QTOF Analysis

Removal of plasma proteins was performed before LC-MS analysis of the plasma metabolites. The plasma samples were thawed on ice and 40 µL of each sample was added into a 96-well Sirocco™ plasma protein filtering plate (#186002448, Waters) containing 180 µL of 90% methanol 0.1% formic acid solution, and the plates were vortexed for 5 min to extract metabolites from the plasma protein precipitate. A 96-well plate for the ultra-performance liquid chromatograms UPLC autosampler (Waters, cat # 186002481) was placed underneath the protein filtering plate and vacuum was applied to the plates (using a manifold) whereby the rubber wells in the Sirocco™ plates opened and the crash solvent including metabolites dripped into the 96-well UPLC plate. When the filtering plates were dry, 180 µL of a 20:80 acetone/acetonitrile solution containing 0.1% formic acid was added to each well to further extract metabolites from the precipitated protein and vacuum was connected until dryness. The solvent was evaporated from the UPLC plates by using a cooled vacuum centrifuge and the dry samples were redissolved in 200 µL milliQ acidic water before analysis. A blank sample (0.1% formic acid) and a standard sample containing 40 different physiological compounds (metabolomics standard) was also added to spare wells to evaluate possible contamination and/or loss of metabolites in the filtering procedure.

Each sample (10 µL) was injected into the UPLC equipped with a 1.7 µm C18 BEH column (Waters) operated with a 6.0 min gradient from 0.1% formic acid to 0.1% formic acid in 20:80 acetone/acetonitrile. The eluate was analyzed in duplicates by TOF-MS (QTOF Premium, Waters). The instrument voltage was 2.8 or 3.2 kV to the tip of the capillary and analysis was performed in negative or positive mode, respectively. In the negative mode desolvation gas temperature was 400 °C, cone voltage 40 V, and Ar collision gas energy 6.1 V; in the positive mode we used the same settings except for collision energy of 10 V. A blank (0.1% formic acid) and the metabolomics standard were analyzed after every 50 samples during the run.

### 3.3. Authentic Standards

L-carnitine, linoleic acid and gamma-linolenic acid were purchased from Sigma Aldrich (Copenhagen, Denmark). 1-acyl LPC(18:1), 1-acyl LPE(18:1), PC(16:0/18:1) and PE(16:0/18:1) were obtained from Avanti Lipids (Alabaster, AL, USA). For the synthesis of acetyl L-carnitine, carnitine acetyltransferase from pigeon and acetyl coenzyme A were purchased from Sigma Aldrich. Acetylation of L-carnitine was performed as described by Bergmeyer *et al.* [32]. The 2-acyl lyso-forms were synthesized with phospholipase A1 from Thermomyces lanuginosus (Sigma Aldrich). Phospholipase A1 hydrolyzes the acyl group attached to the 1-position of PC(16:0/18:1) and PE(16:0/18:1) so that acyl-2 LPC(18:1) and LPE(18:1) were produced. The description of the method has been given by Pete *et al.* [33]. For the chemical verification of identified metabolites, one plasma sample from a rat in the fasted and another from the fed state were spiked with LPC(18:1) and LPE(18:1) individually, before analysis by the procedure outlined above.

### 3.4. Raw Data

The MassLynx^TM^(Version 4.1, Waters, Milford, MA, USA) software collected centroided mass spectra in real time using leucine-enkephalin as a lock-spray standard injected every 10 s to calibrate mass accuracy. Each of the 80 samples was analyzed in duplicates. For negative mode both measurements were included in the data analysis. However, for positive mode 64 sample measurements were excluded, which leaves 65 and 31 sample measurements for fasting and fed states, respectively. The exclusion criterion was based on an instrumental error occurred during analysis. In this case, the outliers had very low intensity due to injection errors.

The software stores data as non-uniform sample data files, each comprised of three vectors; retention time (0–6 min), *m/z* and intensity. The raw data was converted to an intermediate netCDF format with the DataBridge^TM^ utility provided with the MassLynx software.

### 3.5. Software Tools for Data Preprocessing

Raw data was transferred to MarkerLynx^TM^ (Version 4.1, Waters, Milford, MA, USA) directly from MassLynx whereas netCDF files were imported to MZmine [14] and XCMS [15].

The available information regarding the principle of algorithms used in MarkerLynx^TM^, MZmine and XCMS and the selected data preprocessing parameters are shown in electronic Supplementary information 1. The raw data was inspected while selecting the parameters for each software tool. For the peak detection step parameters such as minimum peak width included in MZmine (minimum and maximum peak width included in XCMS) and *m/z* tolerance included in MZmine (ppm in XCMS) were chosen by inspecting the raw data in a 2D sample plot (retention *vs. m/z*). For the alignment step (or peak grouping) TIC of at least 10 samples were overlapped to decide maximum retention time shift between samples. On the other hand, some parameters such as noise level or required peak shape were not straightforward to decide. Thus, at least 10 different parameter settings slightly varying were evaluated for each software tool. The optimum parameters were selected based on the best separation in a PCA scores plot. Deisotoping is performed in MATLAB for XCMS preprocessed data. The final outcome from each software tool is a feature set where each feature is denoted by the mass over charge (*m/z*) ratio and a retention time. The feature sets from the three software tools were transferred to MATLAB for further data analysis.

### 3.6. Custom Methods for Data Preprocessing

**Figure 5:**
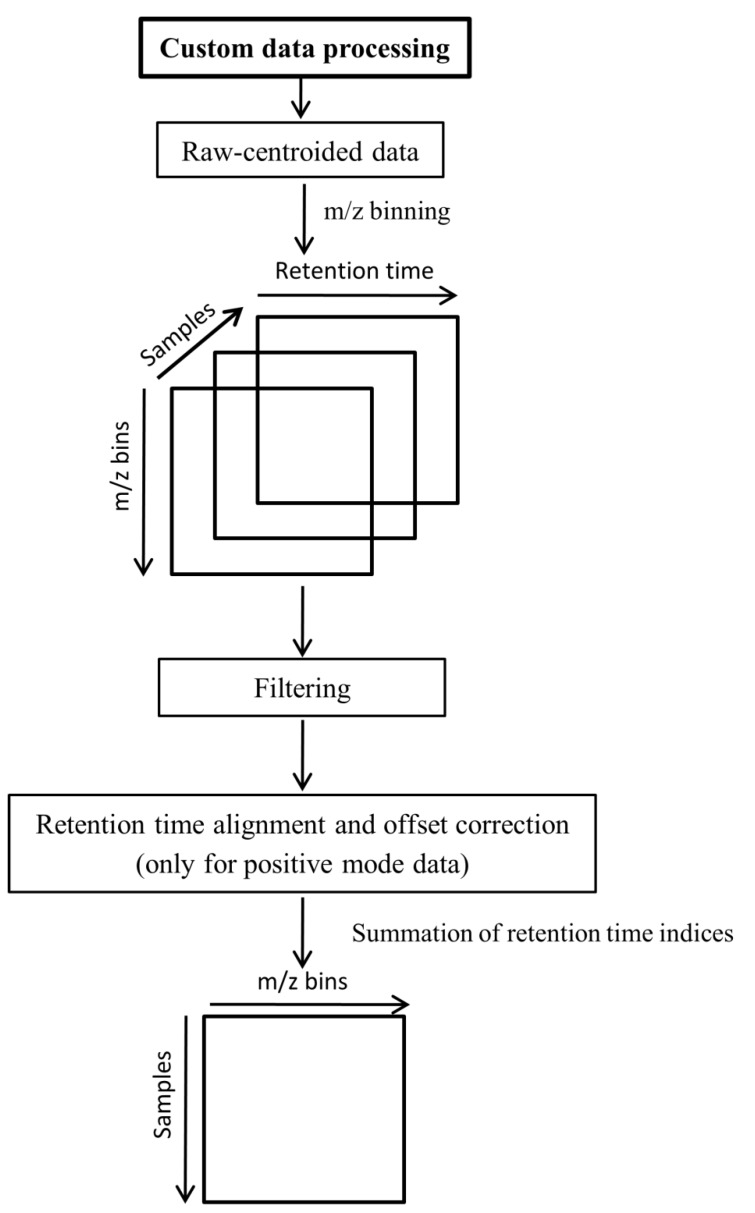
Custom data preprocessing scheme.

An alternative data preprocessing was performed directly on the raw data using MATLAB (Version 7, The Mathworks, Inc., MA, USA). To import netCDF files to MATLAB, the iCDF function [17,34] was employed. The steps of the custom data preprocessing are shown in Figure 5. As the first step, binning was performed on the *m/z* dimension as described by Nielsen *et al.* [17].

Alignment and offset correction were applied only to positive mode data as the instrumental response was observed to be significantly lower during the duplicate runs in the positive mode. To correct for instrumental response differences, prior alignment was performed using ICOshift [35]. The lower response of duplicates was corrected by calculating the difference matrices between each duplicate set, averaging and adding the average difference to the matrix with the lower response. Here it is assumed that the first injection of a sample holds the correct instrumental response whereas its duplicate with lower response is the one being corrected. The effect of this procedure is shown in Supplementary information 2.

A threshold level was applied for the elimination of small peaks/intensities lower than the analytical detection level. Values lower than a certain threshold level were considered as zero. The strategy to define the threshold was as follows: (1) The first median value of the whole dataset (excluding zeros) was calculated; (2) That median was evaluated as a threshold (by the ability of principal component analysis (PCA) score plots to fully separate the fasted *vs.* the fed state (data not shown); (3) The next median was calculated by using only those data from the whole dataset that were higher than the previous median, and again the corresponding PCA scores plot (not shown) was evaluated; (4) This procedure was iterated until an improved separation was achieved by PCA. The threshold levels of the fourth median with the value of 16.17 cps (count per second) in the negative mode and 24.85 cps in the positive mode were selected as adequate.

To enable the application of subsequent two-dimensional data analysis methods, the intensity values of each sample matrix were summed (or collapsed) throughout the retention time index. The resulting data matrix (two-dimensional) is described by samples *vs.m/z* bins (Figure 5) and is also referred to as feature sets throughout this paper.

### 3.7. Data Analysis

The feature sets preprocessed by the three different softwares and the customized method were normalized to unit length and autoscaled. Autoscaling refers to combination of mean centering and unit scaling. 

The PLS_Toolbox (Version 5.3, Eigenvector Research, Inc., MA, USA) was used to implement the data analysis. PCA [36] was applied individually on feature sets obtained from each data preprocessing method for general visualization of discrimination of samples from rats in fasted *vs.* fed state.

PLS-DA is based on the development of a PLS model [37] to predict class membership of a dataset X with a y vector including only 0 and 1 (1 indicates that one sample belongs to a given class). Validation of PLSDA classification models was performed by cross model validation as recommended by Westerhuis *et al.* [18]. 25% of the samples were divided as an independent test set. The remaining samples were cross validated (4-fold) to determine optimal number of latent variables that offers minimum cross validation classification errors. In addition, permutation test is applied with 2,000 random assignments of classes. The test set sample classification errors were evaluated to qualify the classification results.

#### 3.7.1. Variable Reduction

A rough and effective variable reduction procedure was performed specifically during MarkerLynx^TM^ and custom data preprocessing by only keeping a feature if it had a nonzero measurement in at least 80% of the intensity values recorded within one of the sample groups (fasting *vs.* fed in this case); otherwise the feature was removed (80% rule) [38]. Gap filling (or peak finding) algorithms implemented in MZmine and XCMS softwares resulted in few zero entries. However, additional filtering algorithm was enabled in MZmine and XCMS prior to gap filling, which removes any feature if it appears in less than 10 samples (settings are defined in Supplementary information 3).

#### 3.7.2. Variable (Feature) Selection

Further variable selection was performed with PLS-DA. The features or *m/z* bins with larger regression coefficients were considered as more discriminative between fasted and fed states and were regarded as potential biomarkers. Due to the fact that PLS-DA is very prone to overfitting, instead of applying only a single cross-validated PLS-DA model for variable selection on all samples, we performed repeated submodel testing. This implies removing samples randomly (here 10% were taken out at a time), constructing a PLS-DA model on the remaining 90% samples and repeating this 1,000 times. By performing many models the importance of each feature for class separation is tested. The number of latent variables (LV) for each model was determined to minimize the classification errors using cross validation (CV). For each model the features are given a ‘rank’ in the order of their regression coefficients and the final rank of each feature for all the 1,000 submodels were summarized with one number using the median of the 1,000 ranks per feature. This method has the potential of reducing false positives so that the features appearing with higher rank in only a few of the submodels were not considered as markers. We arbitrarily selected the 25 top rank features from each feature set, *i.e.*, those with highest absolute regression coefficient products as potentially representing biomarkers. However, since these features might be daughter ions, adducts, summed ions, *etc.*, we chose here to simply call them ‘markers’ whereas after identification the compounds represented by these markers in the top rank feature sets will be termed ‘biomarkers’. 

### 3.8. Marker Identification

The initial identification of markers was performed according to their exact mass compared with those that were registered in the Human Metabolome Database [39]. Possible fragment ions were investigated by an automated tool using a mol-file format of a candidate compound (MassFragment^TM^, Waters). Further confirmation of candidate biomarkers was obtained by verification of the retention time and fragmentation pattern of an authentic standard (see authentic standards section above). The authentic standards were in some cases selected as one representative of biomarkers belonging to the same chemical compound class, *i.e.*, only one LPC out of a series was confirmed by a standard. Additionally, acyl-1 and acyl-2 LPC(18:1) and LPE(18:1) were spiked into two plasma samples collected in the fed and fasted states, respectively, at a concentration of 0.5 mg/L for a more reliable confirmation.

## 4. Conclusions

We aimed here to explore the effect of four data preprocessing methods on the pattern of final biomarkers for the fasting and fed states in a small rat study. In our custom method, the binning followed by collapsing across retention time gives rise to false positives and negatives. Even so, half of the marker bins selected contained markers detected by at least one of the other softwares.

The less selective peak picking algorithm for Markerlynx^TM^ and the avoidance of peak picking algorithms for the custom method gave rise to detection of some markers that could not be detected by MZmine or XCMS. On the other hand, the gap filling algorithms in MZmine and XCMS improves marker selection because the true signal differences between groups becomes more correct, *i.e.*, in accordance with the raw data.

The selection of proper software parameters based on the specifics of the dataset is the key for obtaining a high quality data analysis, regardless of the applied software. The better parameter setting is a matter of experience and wrong settings may obscure the extraction of relevant information. The use of more than one software and/or the use of several settings during data preprocessing with any softwareare likely to improve marker detection in untargeted metabolomics.

Although the comparison of the selected marker ions from different data preprocessing methods revealed some differences, further chemical identification revealed that they were often just adducts or daughter ions representing the same biomarker compound. Many of the biomarkers identified were chemically closely related so that any of the softwares and procedures applied here could identify biomarkers explaining a major part of the biological processes differing between the fasting and the fed states in our dataset. Thus, all data preprocessing methods agree that specific lipids, carnitines and amino acids are of importance for discriminating plasma samples from the fed and fasting states. Three major lipid classes, LPCs, LPEs and free fatty acids, emerged as discriminative markers in the rats. The high level in the postprandial state of LPCs, generally known to be pro-inflammatory, is interesting and their possible importance for low-grade inflammation in humans should be further explored. L-carnitine and acyl carnitines were also found as important markers and the shift from free to acylated carnitine during fasting might be useful as a marker to follow the switch from postprandial lipid storage to the lipid degradation during fasting. Finally, proline and possibly branched chain amino acids seem to be important amino acid markers that decrease in the fasting state when protein catabolism is necessary for their availability.

## Supplementary Files

Supplementary File 1 DOCX-Document (DOCX, 749 KB)
