# Supplementary material for: The Effect of LC-MS Data Preprocessing Methods on the Selection of Plasma Biomarkers in Fed vs. Fasted Rats
Source: Metabolites. 2012 Jan 18;2(1):77–99. doi: 10.3390/metabo2010077 (PMC3901197; doi:10.3390/metabo2010077)

**Supplementary Information 1.** Principles of algorithms of data preprocessing softwares.

XCMS offers two different peak detection algorithms, *matchedFilter* and *centWave*. The latest developed, *centWave*, was recommended for very complex mixtures which can be represented as plasma in our case. The algorithm first detects the regions of interest in *M/Z* domain based on user defined parameters for mass accuracy (ppm) and maximum and minimum expected chromatographic peak width (peakwidth). Next, chromatographic peaks with different widths were detected using continuous wavelet transform. Finally, features are excluded based on user defined signal to noise ratio (snth). The XCMS alignment algorithm groups peaks together across samples using overlapping *M/Z* bins and calculation of smoothed peak distributions in chromatographic time.

MZmine performs peak detection in two steps. The first step is chromatogram builder, which creates continuous chromatograms for each mass within the user-defined mass range (*M/Z* tolerance) based on mass accuracy of the employed instrument. The width of each peak is determined within the range of the chromatogram limited by the user-defined minimum peak width (min time span) and its absolute height is determined with a restriction on height (min absolute height. Each chromatogram is then deconvoluted using one of the four available algorithms. In this study we applied *local minimum search* for deconvolution of the chromatograms. This algorithm is based on separation of peaks based on their local minima. For alignment MZmine offers linear (*join aligner*) and nonlinear (*ransac peak list aligner*) methods. In this study, limited shifts in retention time favored the use of *join aligner* where its algorithm requires user-defined mass and retention time windows (*M/Z* and retention time tolerance). The algorithm tries to match each peak in a master peak list with the peaks in the sample lists and finds the best match based on the retention time and mass windows.

MarkerLynx as a commercial software is using algorithms which are not publicly revealed and is thus a kind of black box. In the manual it is stated that the software is applying peak detection by the *ApexPeakTrack* peak detection algorithm. MarkerLynx initially determines the regions of interest in the *M/Z* domain based on mass accuracy (mass tolerance). The ApexPeakTrack algorithm controls peak detection by peak width (peak width at 5% height) and baseline threshold (peak to peak baseline ratio) parameters which can be either set by user or calculated automatically. The algorithm finds the inflection points (peak width at 5% height), local minima and peak apex to decide peak area and height. It also calculates the baseline noise level using the slope of inflection points. Compared to peak detection algorithms of other softwares, the ApexPeakTrack algorithm produces a much higher number of peaks, so an additional peak removal step (denoted by user defined peak intensity threshold and noise elimination level parameters) is conjugated to the alignment algorithm by its developers. The basic principle of peak removal is described in the accompanying materials: “If a peak is above threshold in one sample and if it is lower than threshold in another sample it lowers the threshold for that sample until it reaches the noise elimination level”. The MarkerLynx alignment algorithm performs alignment of peaks across samples within the range of user-defined mass and retention time windows. (MassLynx (Waters, Milfold, MA, USA)).

**Supplementary Information 2.** TIC of the samples obtained from positive mode (a) before and (b) after alignment and normalization using the average difference between replicates that are shifted. The two small inserts below each graph show zoomed parts at two retention time intervals.


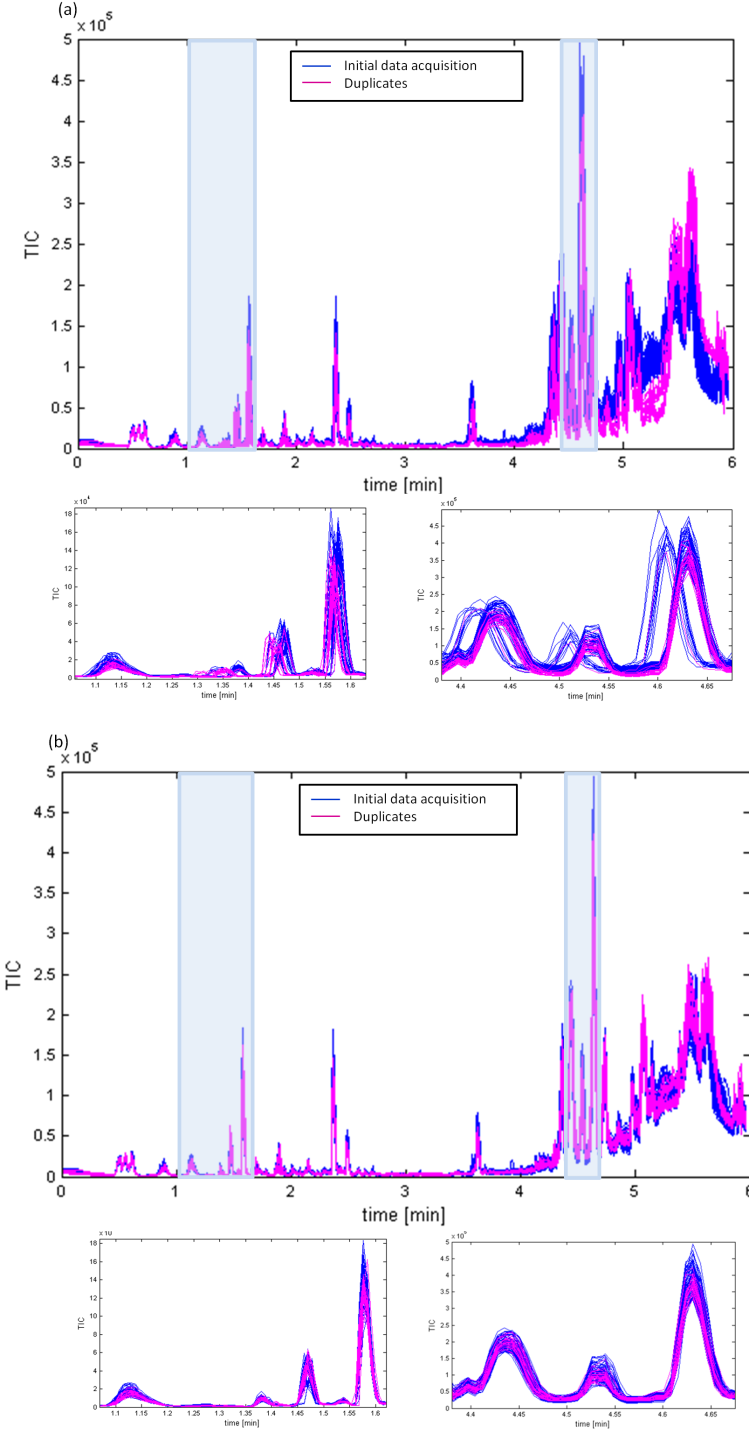


**Supplementary Information 3.** Data preprocessing steps and its parameters settings for MarkerLynx, MZmine and Custom preprocessing (pos: positive mode data; neg: negative mode data).

|  | **MarkerLynx** | **MZmine** | **XCMS** | **Custom** |
| --- | --- | --- | --- | --- |
| Peak Detection | ApexPeakTrack  Peak width at 5% height = default  Peak-to-peak baseline ratio = default  Noise elimination = 4  Intesity threshold = 30 (neg); 60 (pos) | Highest data Point  Min time span = 0:01  Min absolute height = 20 (neg); 60 (pos)  *M/Z* tolerance = 0.04 (neg); 0.03 (pos)  Local minimum search  Min RT range = 0:01  Min absolute height = 30 (neg) − 60 (pos)  Min peak top/edge = 1.5 | centWave  ppm = 30 (neg); 40 (pos)  peakwidth = (2,10)  snth = 4 (neg); 5 (pos)  prefilter = c(1,40) (neg); c(1,80) (pos) | No peak detection |
| Normalization | ✓ | - | - | - |
| Deisotoping | ✓ | ✓ | ✓ | ✓ |
| Alignment | *M/Z* window = 0.05  r/t window = 0.05 | Join aligner  *M/Z* tolerance = 0.05  RT tolerance = 0:03 | Group  bw = 4  mxwid = 0.05  Retcor  obiwarp  profStep = 0.1 | ICOshift (pos) |
| Filtering | 80 % rule | Peak list row filter  Min peaks in a row = 10  Duplicate peak filter  *M/Z* tolerance = 0.01  RT tolerance = 0.01 | Implemented in previous group function  minfrac = 0.1 | 80 % rule |
| Gap filling | - | Peak finder  *M/Z* tolerance = 0.02  RT tolerance = 0:02 | Fillpeaks | - |

**Supplemantary Information 4.** Number of features extracted from each data processing method.

|  | **MarkerLynx** | | **MZmine** | **XCMS** | **Custom** | |
| --- | --- | --- | --- | --- | --- | --- |
| NEG | Before 80% rule: | 3780 | 1501 | 1562 | Before 80% rule: | 9500 |
|  | After 80% rule: | 1852 |  |  | After 80% rule: | 3700 |
| POS | Before 80% rule: | 6065 | 3272 | 2714 | Before 80% rule: | 9500 |
|  | After 80% rule: | 2981 |  |  | After 80% rule: | 3894 |

**Supplemantary Information 5.** PCA scores plot of MarkerLynx (A), MZmine (B), XCMS (C) custom preprocessed (D) positive mode data.


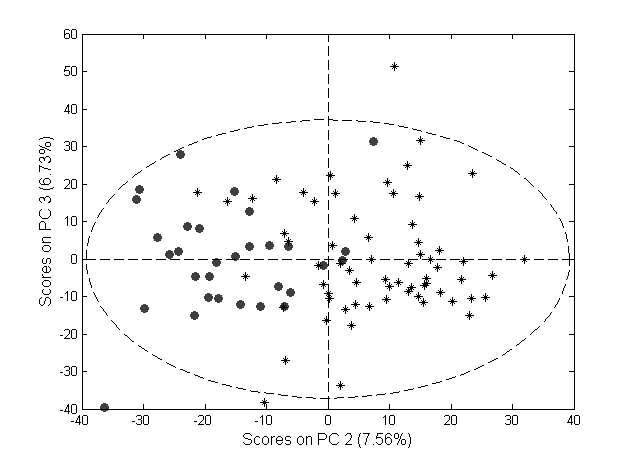


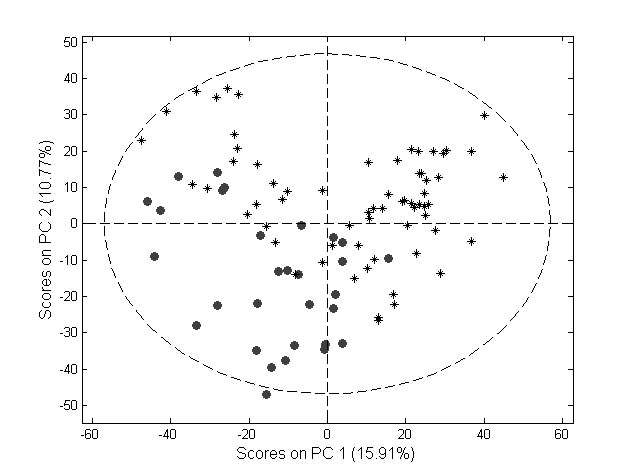


**Supplemantary Information 5.** *Cont*.


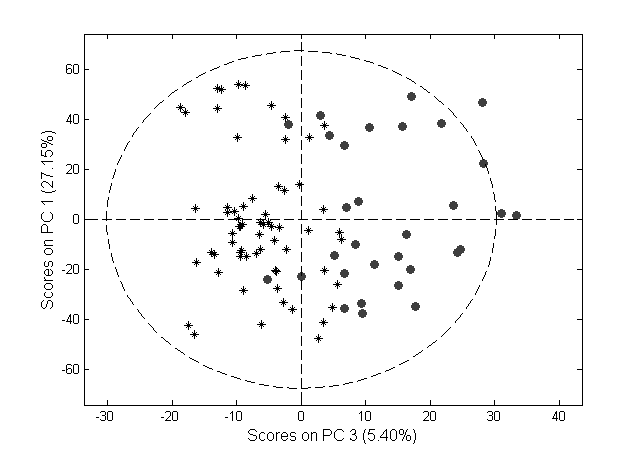


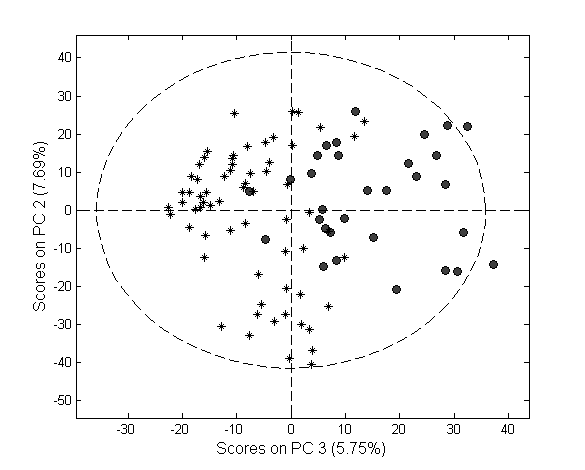


**Supplementary Information 6.** PLSDA model classification error rates of test sets.

|  | **Classification Error Rates** | | | |
| --- | --- | --- | --- | --- |
| Negative mode data | 0.00 | 0.01 | 0.01 | 0.01 |
| Positive mode data | 0.00 | 0.02 | 0.02 | 0.02 |

**Supplemantary Information 7.** Classification error rates based on cross model validation predictions of the correct classes (red arrow) and permuted class labels (black bars) for MarkerLynx (1), MZmine (2), XCMS (3) custom preprocessed negative (A) and positive mode (B).

**B3**

**B4B2**

**B2B2**

**B1B2**

**A4**

**A3**

**A1**

**A2**

**Supplemantary Information 8.** The chromatogram of *M/Z* bin = 819.6, from 4.3 to 4.5 min. The peaks are detected as two separate features by the other softwares (Peak no: 42 and 33, positive mode). Red tracks, fasting state; black tracks fed state.

**Supplemantary Information 9.** (**A**) MZmine and (**B**) MarkerLynx recorded peak heights of samples in fasted and fed groups for marker number 42. The difference between the two groups in (B) is inflated as MarkerLynx recorded the signal as zero for many of the samples in fed group. Thus this marker has lower rank in MarkerLynx.

(**A**)

**
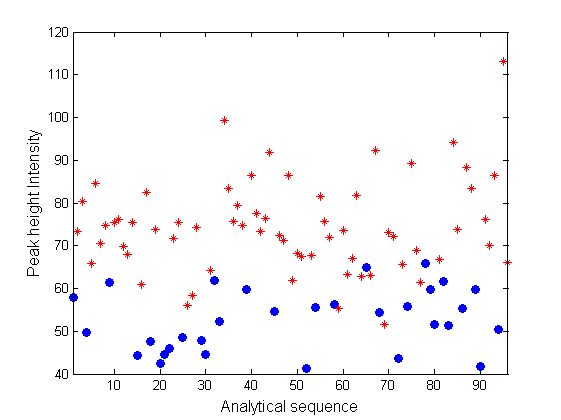
**


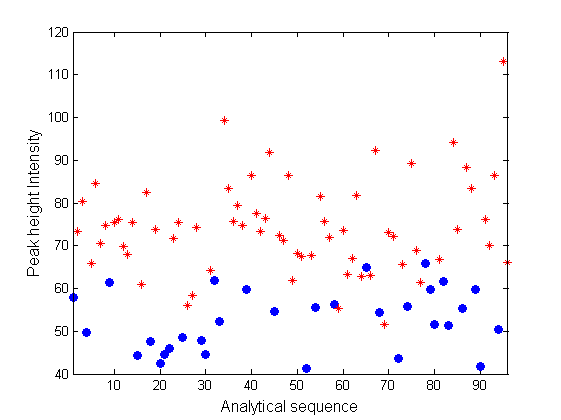

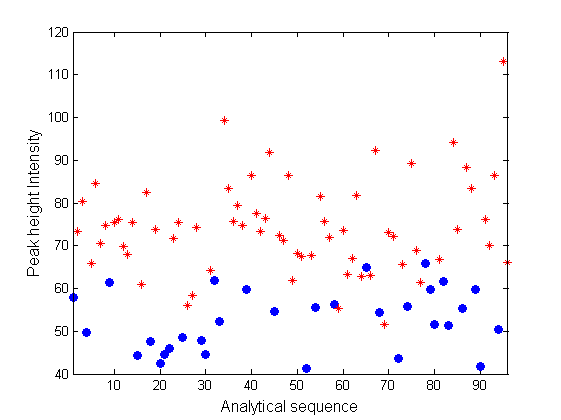


Fasted

Fed

**Supplemantary Information 9.** *Cont*.

(**B**)


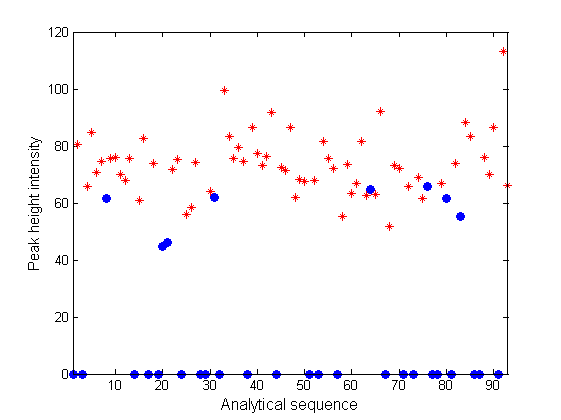


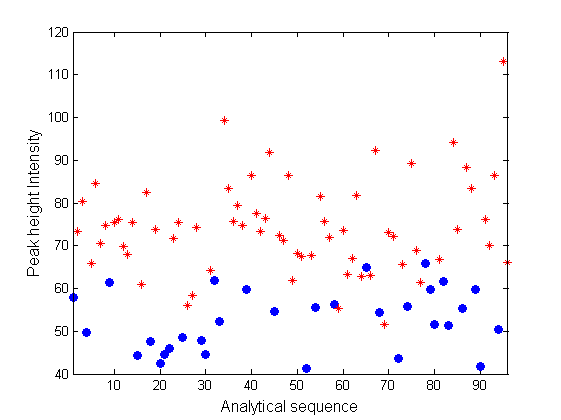

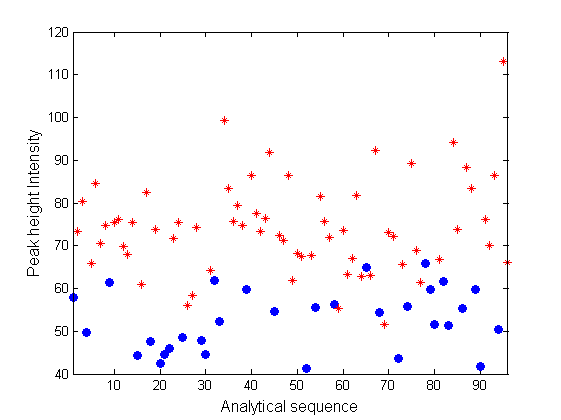


Fasted

Fed

**Supplemantary information 10.** (A) MZmine and (B) MarkerLynx recorded peak heights of samples in fasted and fed groups for marker number 38. The difference between the two groups in (B) is deflated as MarkerLynx recorded the signal as zero for some of the samples in the fed group. Thus this marker has higher rank in MarkerLynx.

(**A**)


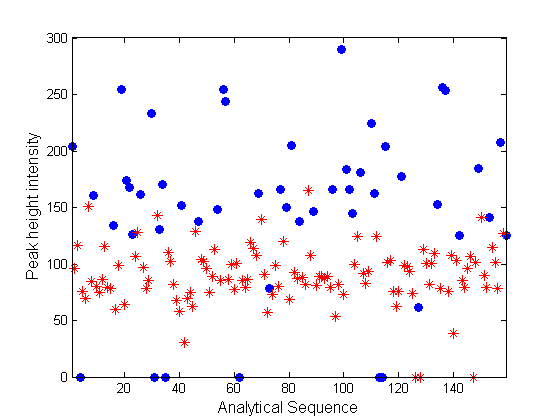


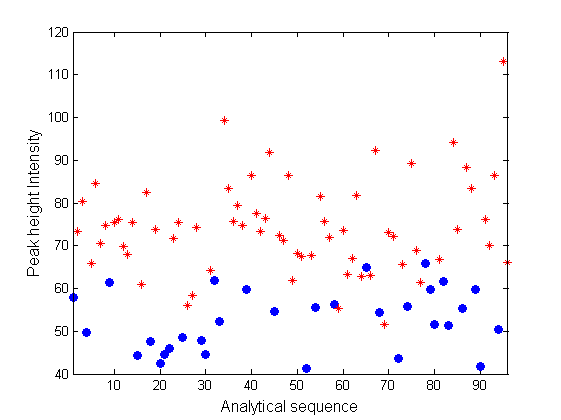

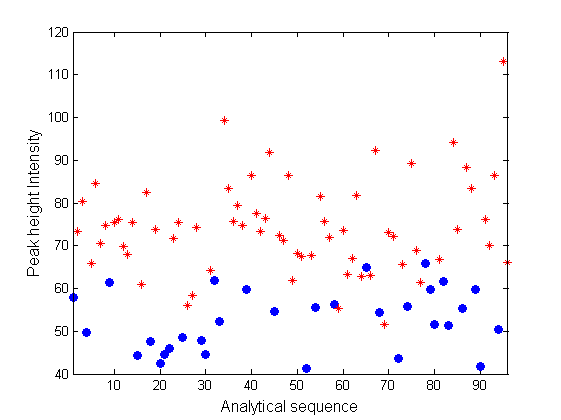


Fasted

Fed

**Supplemantary Information 10.** *Cont*.

(**B**)


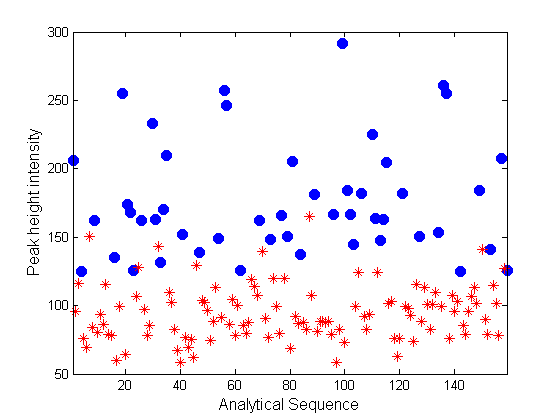


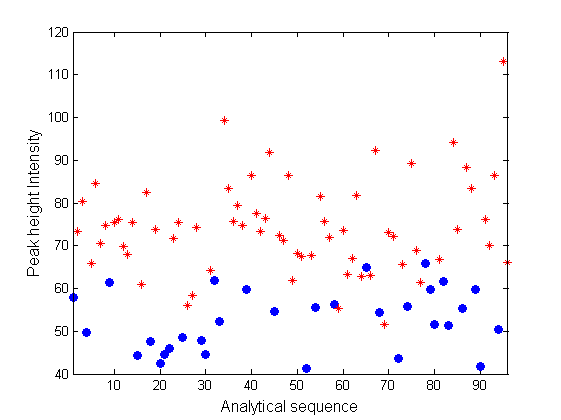

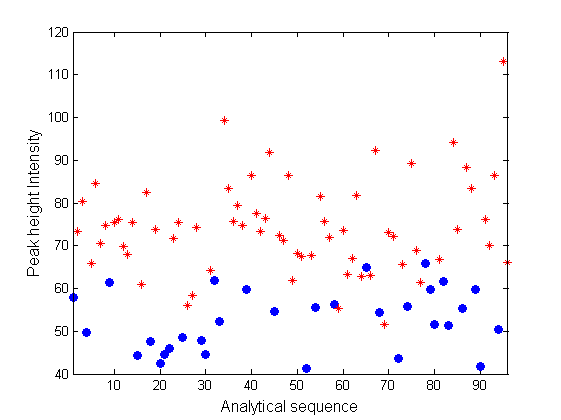


Fasted

Fed

**Supplemantary Information 11.** XIC of 1-acyl and 2-acyl LPC(18:1) detected in positive mode ionization. The panels in sequence from top to buttom show the extracted ion chromatogram for *M/Z* 522.358 of 1. an authentic rat plasma sample; 2. The same sample spiked with 1-acyl LPC(18:1); 3. The sample spiked with 2-acyl LPC(18:1); 4. A 1-acyl LPC(18:1) standard; 5. A 2-acyl LPC(18:1) standard.


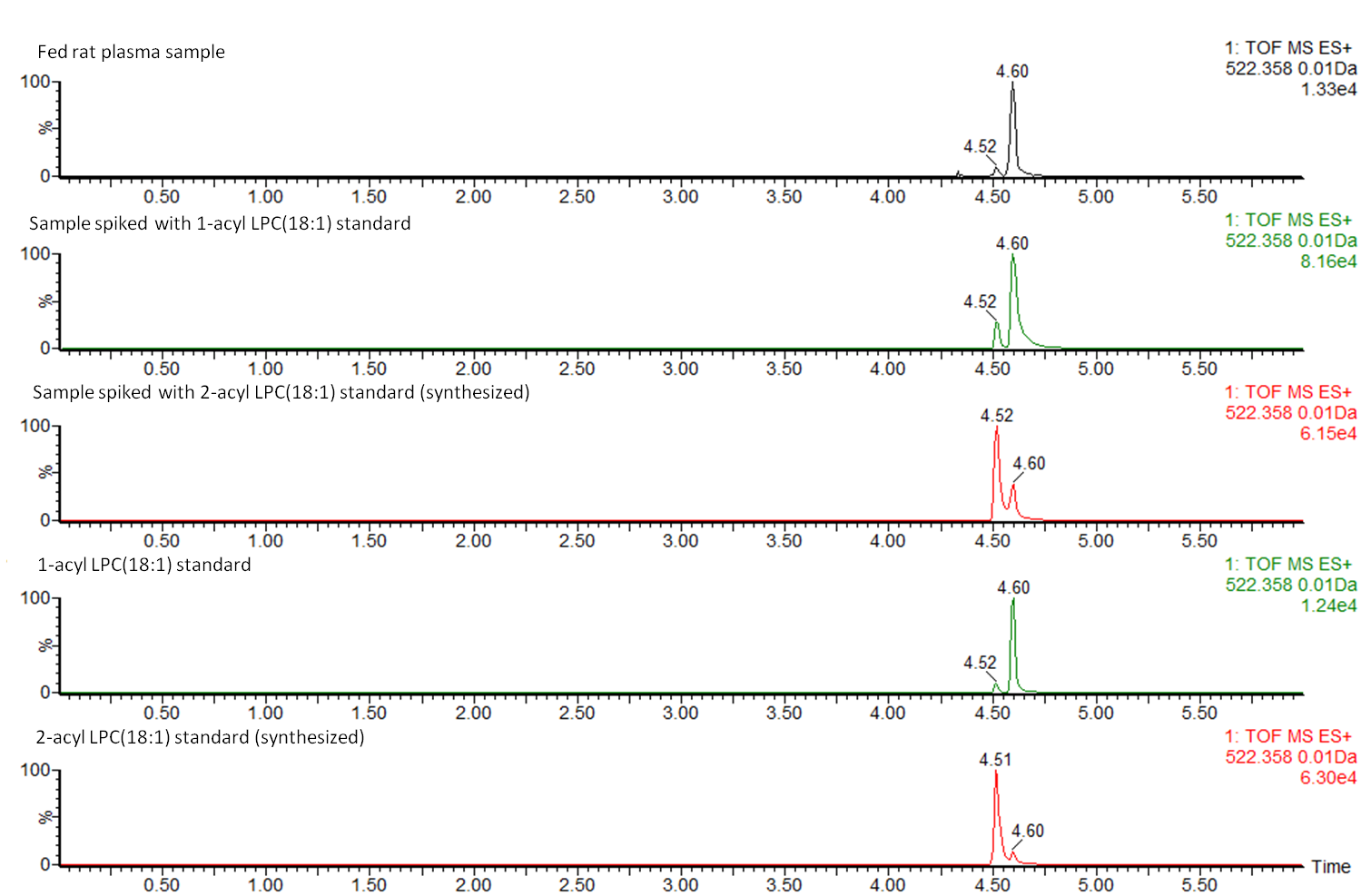


**Supplemantary Information 12.** Heatmap comparing the importance of metabolite based on four different data preprocessing tools. (MarkerLynx, MZmine, XCMS and Custom data processing) for (**a**) negative and (**b**) positive mode data. Each row represents the rank (importance) of a marker for four different methods (from Table 1 or 2, 3rd column). The markers selected had a rank below 25 with at least one of the four methods. The markers were sorted in ascending rank order of MarkerLynx. (red: rank 1-25; orange: rank 26-50; yellow: rank>50; black: not detected).

(**a**)


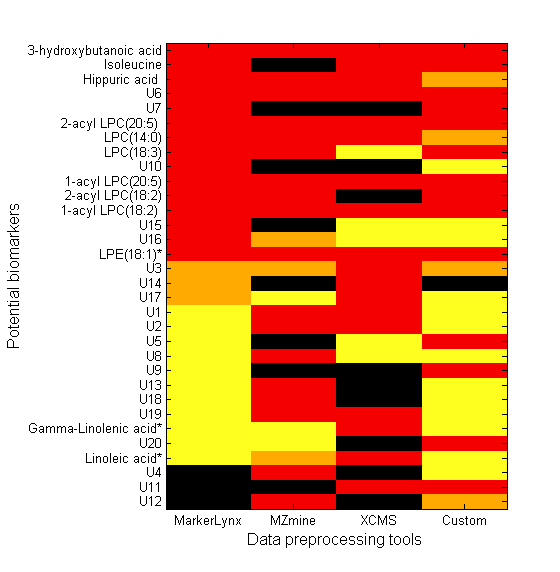


**Supplemantary Information 12.** *Cont.*

(**b**)


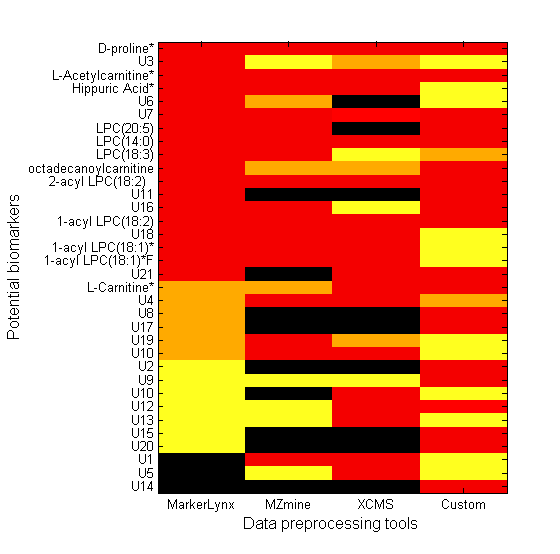

Supplement: Supplementary File 1 — DOCX-Document (DOCX, 749 KB) [file metabolites-02-00077-s001.docx]
